# Surgical treatment and a unique management of rostral mandibular fracture with cerclage wire in a horse

**Published:** 2015-06-15

**Authors:** Hadi Naddaf, Soroush Sabiza, Narges Kavosi

**Affiliations:** *Department of Clinical Sciences, Faculty of Veterinary Medicine, Shahid Chamran University of Ahvaz, Ahvaz, Iran.*

**Keywords:** Cerclage wire, Fracture healing, Horse, Mandible fracture

## Abstract

A 3-year-old Arabian colt was presented for a major gingiva wound at the right rostral part of mandible. After clinical assessments, rostral mandibular fracture was determined. Stabilization of fractured region was achieved via cerclage wire application under general anesthesia. Fixation wires were left in place for 6 weeks. A 3 -month follow up revealed complete fracture healing. The purpose of this case report was to give clinical information about rostral mandibular fractures and treatment of these fractures and nutrition protocol in a horse, as this fracture is of the most common type of jaw fracture sustained by young horses.

## Introduction

Fractures of the rostral mandible are the most common type of jaw fracture sustained by young horses.^1^ Fracture configuration differs between individuals, but in essence the injury comprises a partial avulsion of one or more incisor teeth with a variable amount of associated bone. Fractures range from simple loss or loosening of a single (usually corner) incisor tooth and its labial alveolar bone plate, to deep fractures involving a portion of mandible housing several incisor teeth.^2^^,^^3^ The fracture line usually describes an arc linking the interdental space of the mandibular bar on one side and exiting between any two mandibular incisor teeth. The fractured portion of the mandible is displaced ventrally, with a resulting open gingival wound on the floor of the mouth communicating with the open fracture line. Occasionally the injury will involve some compression of fracture fragments caudally or dorsally but ventral distraction is the norm. Fracture of the actual incisor teeth is unusual, and only rarely are bilateral fractures encountered.^4^^,^^5^ The purpose of this case report was to give clinical information about rostral mandibular fractures and treatment of these fractures and nutrition protocol in a horse, as this fracture is of the most common type of jaw fracture sustained by young horses.

## Case Description

**Fig. 1:**
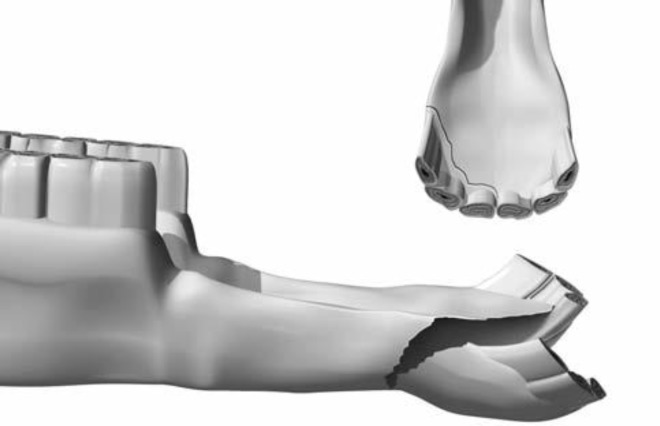
Rostral mandibular fracture in a horse.^7^

**Fig. 2:**
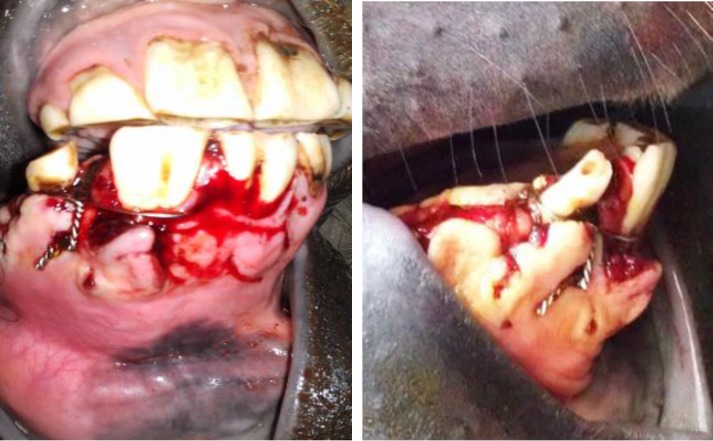
Cerclage wire for rostral mandibular fracture in a 3-year-old Arabian colt.

**Fig. 3:**
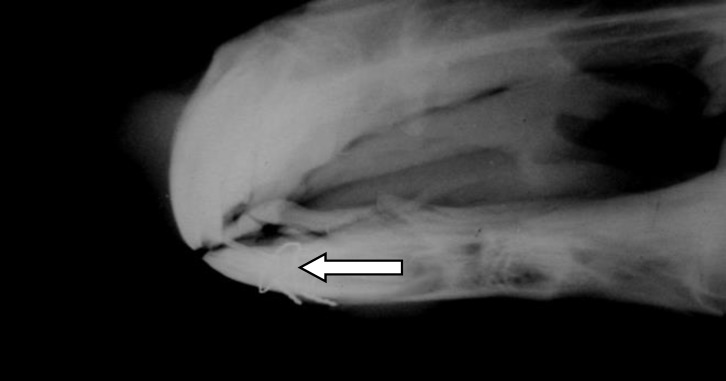
Radiography of  cerclage wire (arrow) for rostral mandibular fracture in a 3-year-old Arabian colt.

A 3-year-old Arabian colt was presented to the veterinary hospital of Shahid Chamran University of Ahvaz with a major gingival wound at the right rostral part of mandible. According to information obtained from the owner, the horse had fought with the other horse and it caused oral cavity bleeding. A rostral mandibular fracture and of course gingival wound, were detected following clinical examinations (Fig. 1). Initially, vital signs (heart rate, respiratory rate, rectal temperature) and complete blood count parameters were evaluated in normal ranges and these factors stated general health of the patient prior to surgical operation.  Vital signs were: Heart rate: 30 beat per min, respiratory rate: 12 breaths per min, rectal temperature: 38.5 ˚C. Complete blood count revealed PCV of 35.9%, segmented neutrophil of 65.0%, lymphocyte of 30.0%, eosinophil of 5.0%. The horse initially was sedated by intravenous (IV) 1.1 mg kg^-1^ of xylazine, (Alfasan, Woerden, The Netherlands).^6^ Then, a 14 gauge angio-catheter were fixed in right jugular vein. Anesthesia was induced using 2.2 mg kg^-1^ of ketamine (Alfasan, Woerden, The Netherlands) plus 0.2 mg kg^-1 ^of diazepam (Chemidarou Pharmaceutical Co., Tehran, Iran) through the catheter and 1.1 mg kg^-1^, IV ketamine was intermittently applied according to the horse’s reactions for the constancy and duration of the anesthesia.^6^ The oral cavity and fractured bone ends were washed with sterile saline to remove debris and saliva and then were prepared for operation by washing with diluted povidone iodine. A 1.2 mm pin was used to create two holes for passing cerclage wire on the fractured area. The pin was placed on the bone just below the first and second incisive tooth, and directed from the lateral to medial side of the rostral mandible and to the rostral sublingual oral mucosa. When first hole was created, the pin was removed and a 14 gauge needle was inserted in first hole from the lateral to medial side of the rostral mandible. Cerclage wire was placed in 14 gauge needle. Wire was passed through the needle from the medial to lateral side of the rostral mandible. Then, the other hole was created similarly 2 cm distal to the first hole and the cerclage wire was passed the same way. The wire ends were twisted at the ventral side of rostral mandible just below the mandibular incisive teeth for fracture reduction. The sharp ends of wire knots were bent to prevent any soft tissue irritation (Figs. 2 and 3).

Also, methods of anchoring wire caudal to the incisors was used which anchor the wires to the canine teeth in horses.^7^ As it can be easily understood, solid meal and severe movement of jaws cause delay union, a series of proceedings were done as postoperative management as shown in Table 1.

**Table 1:** Postoperative management of the rostral mandibular fracture in a 3-year-old Arabian colt.

**Postoperative managements**	
**Antibiotic therapy**	11 mg kg^-1^ Cefazoline every 12 hours for 7 days IV. 20 mL gentamicin (50 mg mL^-1^) every 12 hours for 7 days IV.
**Anti-inflammatory drug**	Ketoprofen (3 mg kg^-1^) daily for 3 days intramuscular.
**Fluid therapy**	Ringer’s solution (10 L, IV) daily for 5 consecutive days. 50% dextrose (250 mL, IV) daily for 5 consecutive days.
**Nutrition**	First 5 days (Liquid diet): 500 g ORS in 20 L water + 250 g sugar, daily, orally. Next 20 days (Semi solid diet): Such as combination of bran + water.
**Supportive treatment**	IV administration of Phosphorus + B12 (30 mL) and repeat it a week later with the same dose and route.
**Considerations**	To remove probable residue of diet in surgical region, daily lavage of the oral cavity by normal saline. Wire removal after 6 weeks.

**Outcome**

Very rapid gingival wound healing was observed within 10 days following the fixation. A 3-month follow up revealed complete fracture healing.

## Discussion

The objectives of the surgical treatment of mandibular fractures are to restore normal occlusion and provide stability that can support fracture healing and allow normal eating and drinking simultaneously.^8^ These fractures can usually be evaluated without radiography, and most can be reduced and stabilized with interdental wiring.^9^ Fractures of the rostral portion of the mandible or maxilla/ premaxillae in the interdental space most commonly occur from kicks from other horses and are usually transverse. Common techniques used to repair these fractures include lag-screw fixation, application of an intraoral or extraoral acrylic prosthesis, and dynamic compression plating and wiring.^9^ Very rapid gingival wound healing is due to perfect local vascularization of the gingiva.^5^ Although, interdental wire loosening or failure was observed in 22% of the horses.^1^ Several complications such as tooth loss, malocclusion, osteomyelitis, sequestration, as well as chronic discharge are reported.^5^ However, the technique we used in this study had no such complications. Fixation materials were left in place for six weeks. Although complications in horses with fractures of the mandible and maxilla are common, long term prognosis for functional and cosmetic outcome are favorable when repaired with cerclage wire.^10^ Avulsion fractures of the incisors are easily amenable to repair by tension band wiring alone in the standing or anaesthetised patient.^7^ This report described surgical treatment of rostral mandibular fracture with cerclage wire in an Arabian colt and suggested postoperative management protocol for clinicians.
